# Tailoring Targeted Temperature Management in Comatose Out-of-Hospital Cardiac Arrest Survivors: A Retrospective Analysis Based on the rCAST Score Classification

**DOI:** 10.3390/jcm14113931

**Published:** 2025-06-03

**Authors:** Hyojeong Kwon, Hanna Park, Dongju Kim, Sang-Min Kim, June-Sung Kim, Youn-Jung Kim, Won Young Kim

**Affiliations:** Department of Emergency Medicine, Asan Medical Center, University of Ulsan College of Medicine, Seoul 05505, Republic of Korea; hyojeong1214@gmail.com (H.K.);

**Keywords:** out-of-hospital cardiac arrest, post-cardiac arrest syndrome, targeted temperature management, neurological outcome, risk stratification, rCAST

## Abstract

**Background/Objectives**: Stratifying post-cardiac arrest survivors based on the likelihood of good neurologic outcomes can guide the decision for targeted temperature management (TTM). This study aimed to compare the impact of TTM on neurological improvement among comatose out-of-hospital cardiac arrest (OHCA) survivors stratified by the revised post-cardiac arrest syndrome for therapeutic hypothermia (rCAST) score. **Methods**: This retrospective observational cohort study was conducted from February 2018 to April 2023 at the emergency department. We calculated the rCAST score immediately after the return of spontaneous circulation in adult patients and compared neurological outcomes at discharge for TTM based on the severity classification of the rCAST score (low: ≤5.5; moderate: 6.0–14.0; high: ≥14.5). We utilized inverse probability of treatment weighting (IPTW) analysis to adjust for selection bias and potential confounding factors between the TTM and non-TTM groups. **Results**: Among 300 comatose OHCA survivors, the proportions of patients with good neurological outcomes at discharge were 60.7% (17/28), 38.9% (56/144), and 2.3% (3/128) in the low, moderate, and high-severity rCAST groups, respectively. With increasing severity of the rCAST, the absolute difference in the proportion of patients with good neurological outcomes decreased between those who underwent TTM and those who did not (68.0% vs. 0.0%; *p* = 0.023, 45.2% vs. 27.5%; *p* = 0.037, and 3.5% vs. 0.0%; *p* = 0.221, respectively). After adjusting using IPTW, TTM was associated with good neurologic outcomes in the moderate-severity group (odds ratio, 2.31; 95% confidence interval, 1.09–4.91; *p* = 0.029). **Conclusions**: This study suggests that TTM may offer specific benefits for certain groups of OHCA survivors. Further research is needed to refine risk stratification tools for improved patient selection.

## 1. Introduction

Out-of-hospital cardiac arrest (OHCA) is a major global public health issue with high mortality and poor neurological outcomes. Early interventions during the prehospital phase, including bystander cardiopulmonary resuscitation (CPR) and early defibrillation, are key determinants of survival. Recent improvements in emergency response systems and broader access to public defibrillators have modestly improved outcomes. In addition, patient characteristics such as age and residential setting, as well as the dose of adrenaline administered during resuscitation, have also been shown to influence the likelihood of return of spontaneous circulation (ROSC) [1].

Neurological injury following cardiac arrest remains a significant concern despite advances in resuscitation techniques [2]. Although targeted temperature management (TTM) is a potential neuroprotective intervention to improve neurologic outcomes in out-of-hospital cardiac arrest (OHCA) survivors [3,4,5,6], recent studies have reported that TTM was not associated with improved overall survival or neurological outcomes and increased risk of arrhythmias [7]. Nevertheless, the effectiveness of TTM may vary among different patient subgroups [8], emphasizing the importance of accurate risk stratification tools to identify those who would benefit most from this intervention [9].

The revised post-cardiac arrest syndrome for therapeutic hypothermia (rCAST) score is a recently developed scoring system aimed at predicting the neurological outcomes in OHCA survivors treated with TTM [10]. It can be derived using five straightforward clinical factors (initial rhythm, witnessed status and time until ROSC, serum pH, serum lactate, and motor score of the Glasgow coma scale [GCS], which can be assessed prior to the patient’s admission to the intensive care unit (ICU) (Supplementary Table S1). The rCAST score provides a quantitative measure of severity and classifies OHCA survivors into three grades (low-, moderate-, and high-severity groups). Thus, determining the relationship between the ischemic injury severity by rCAST score and the impact of TTM on neurological outcome has the potential to guide clinical decision-making regarding the implementation of TTM.

This study aimed to compare the impact of TTM on neurological outcomes at discharge in comatose OHCA survivors stratified by the rCAST score. By evaluating the relationship between TTM and neurological outcomes in the severity classification of the rCAST score, we can gain valuable insights into the optimal utilization of TTM in specific subgroups of OHCA patients.

## 2. Materials and Methods

### 2.1. Study Design and Population

**Figure 1:**
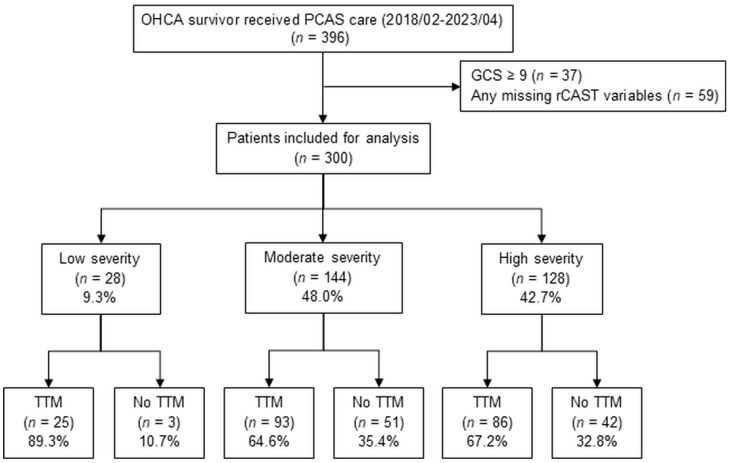
Study flow chart. Abbreviations: OHCA, out-of-hospital cardiac arrest; PCAS, post-cardiac arrest syndrome; GCS, Glasgow coma scale; rCAST, revised post-cardiac arrest syndrome for therapeutic hypothermia; TTM, targeted temperature management.

This single-center, retrospective study was conducted using a prospectively enrolled registry of OHCA patients admitted to Asan Medical Center, a university-affiliated teaching hospital in South Korea. Relevant data from all patients who present to the emergency department (ED) with cardiac arrest have been systematically collected and managed since 2010 [11]. All adult patients (≥18 years) who experienced successful resuscitation following OHCA and were consecutively admitted to the hospital from February 2018 to April 2023 were extracted from the registry. Patients were excluded if they had missing data required to calculate the rCAST score or had a GCS score of 9 or higher, indicating preserved consciousness and ineligibility for TTM. The detailed process of patient selection, inclusion, and exclusion is illustrated in Figure 1. The TTM group included patients who received temperature management based on standard post-cardiac-arrest care criteria, specifically those who remained unresponsive after ROSC with a GCS score of less than 9 [12]. TTM was applied based on a standardized institutional protocol. Surface cooling system with circulating water and hydrogel pads in conjunction with computerized temperature control unit was the first-line method. If unavailable, water-circulating blankets or endovascular cooling methods were used as alternatives. Core temperature was primarily measured via the esophagus, with bladder or rectal sites used when necessary. The target temperature (typically between 33 °C and 36 °C) was maintained for at least 24 h in accordance with international guidelines. TTM was initiated as early as possible, ideally within 6 h after ROSC. Rewarming was conducted gradually at a rate of approximately 0.25 °C per hour until 36.5 °C, followed by controlled normothermia maintained until 72 h after ROSC. In certain cases, modifications were made based on the attending physician’s clinical judgment, such as extending the duration of TTM or using alternative cooling methods, depending on device availability or patient-specific factors. The decision to administer sedatives and analgesics was made by the attending intensivist in accordance with institutional protocols. Neuromuscular blocking agents were used to control shivering or ventilator–patient dyssynchrony. In the non-TTM group, defined as the control group, we included patients who, despite meeting the TTM criteria, did not receive TTM due to non-clinical factors, such as limitations in hospital staffing or ICU bed availability. These factors were more common during the COVID-19 pandemic, although TTM was still applied whenever feasible in accordance with institutional protocols. The analyzed patients were categorized into three groups by rCAST score: low, 0–5.5; moderate, 6–14; and high, 14.5–18.5 [10]. The study protocol was approved by the Institutional Review Board of our institution, and the requirement for informed consent was waived due to the retrospective nature of the study (No. 2023-0926).

### 2.2. Data Collection

The following demographic and clinical information was extracted from the registry, and any missing variables were collected by reviewing the electronic medical records: age, sex, medical history, details of arrest (etiology of arrest, witnessed arrest, bystander CPR, initial rhythm, and duration of resuscitation), laboratory variables of the rCAST score (initial pH and initial lactate), and neurological evaluation after ROSC (pupillary light reflex [PLR] and score of the motor scale of GCS). The duration of resuscitation included the duration of bystander CPR if present. The primary outcome was neurological outcomes at hospital discharge. Good neurological outcomes were defined as a Cerebral Performance Category (CPC) score of 1–2, while poor neurological outcomes were defined as a CPC score of 3–5.

### 2.3. Statistical Analysis

Continuous variables were described using either the mean and standard deviation or the median and interquartile range, depending on the normality of their distribution assessed by the Kolmogorov–Smirnov test. Categorical data were presented as absolute numbers and percentages. Baseline demographics and clinical characteristics were compared across the three rCAST severity groups, as well as between the TTM and non-TTM groups. Continuous variables were analyzed using the Mann–Whitney U test or Student’s *t*-test depending on the normality of distribution. Categorical variables were compared using the Chi-square test when expected cell counts were sufficient or Fisher’s exact test when expected counts were less than 5. A receiver operating characteristic curve with the area under the curve (AUC) was used to determine the predictive accuracy of the rCAST for poor neurological outcomes. Univariate logistic analysis was conducted to assess the association of each variable with good neurological outcomes at discharge. Variables with a *p*-value < 0.1 in univariate analysis were considered for entry into the multivariable logistic regression analysis using a backward elimination method. The logistic regression analysis outcomes were presented as odds ratios (OR) and 95% confidence intervals (CI). For supplementary analysis, patients were classified into pre-pandemic and pandemic periods to compare baseline demographic and clinical characteristics. The pandemic period was defined from 30 January 2020 to 5 May 2023, based on the World Health Organization’s declaration and termination of the Public Health Emergency of International Concern [13]. Additionally, survival to discharge and good neurological outcomes at discharge were compared between the TTM and non-TTM groups within each rCAST severity category. To adjust for selection bias and potential confounding factors between the TTM and non-TTM groups in the moderate-severity rCAST group, inverse probability of treatment weighting (IPTW) analysis. First, propensity scores were estimated using a logistic regression model that included age, sex, arrest etiology, witnessed status, bystander CPR, duration of resuscitation, past medical history, and PLR. These variables were selected based on their clinical relevance to post-cardiac arrest prognosis and TTM decision-making. Next, inverse probability weights were calculated for each patient: the inverse of the propensity score for those who received TTM and the inverse of one minus the propensity score for those who did not. To assess whether IPTW achieved an adequate balance between the groups, standardized mean differences (SMDs) were calculated for all covariates before and after weighting. An SMD < 0.1 was considered indicative of adequate covariate balance. A two-tailed *p*-value of <0.05 was considered statistically significant. All statistical analyses were performed using IBM SPSS Statistics for Windows, Version 21.0 (IBM Corp., Armonk, NY, USA).

## 3. Results

From February 2018 to April 2023, 396 adult OHCA survivors were admitted through the ED for post-cardiac arrest care (Figure 1). A total of 300 patients were included in the final analysis after excluding 37 patients with a GCS ≥ 9 and 59 patients with any missing rCAST variables. Upon stratification based on the severity of rCAST, the low, moderate, and high groups consisted of 28 (9.3%), 144 (48.0%), and 128 (42.7%) patients, respectively. Among them, TTM was performed in 25 (89.3%) of the low group, 93 (64.6%) of the moderate group, and 86 (67.2%) of the high group.

**Figure 2:**
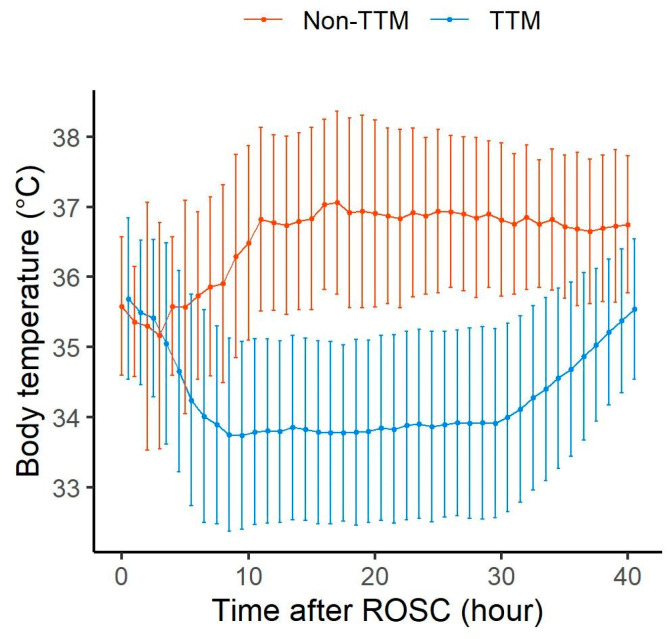
Temperature curves of the TTM and non-TTM groups. The temperature curves show the means, and the error bars indicate ± standard deviation. Abbreviations: TTM, targeted temperature management; ROSC, return of spontaneous circulation.

There were no significant differences in baseline demographic characteristics, clinical information related to cardiac arrest, or the patients’ severity (as measured by Sequential Organ Failure Assessment (SOFA) score at ICU admission) between the TTM group and the non-TTM group (Supplementary Table S2). The TTM groups targeted a temperature between 33 °C and 36 °C, and all patients reached the target temperature. The temperature changes over time in the TTM and non-TTM groups are shown in Figure 2. From the time of ROSC up to 3 h, there was no temperature difference observed between the TTM group and the non-TTM group. However, starting from 4 h after ROSC, there was a significant difference in temperature between the TTM group and the non-TTM group. Baseline demographics and clinical information during the pre-pandemic and pandemic periods are presented in Supplementary Table S3. Most variables were comparable between the two periods.

Table 1 shows the baseline demographics and clinical characteristics of the patients. The median age was 65.5 years, and there was no significant difference in age between each rCAST severity group (*p* = 0.829). The proportion of males (*p* = 0.526) and the frequency of bystander CPRs (*p* = 0.948) did not differ by group. The PLR evaluated after ROSC showed significant differences between each group (both *p* < 0.001). There were differences in survival to discharge (23 [82.1%], 91 [63.2%], and 33 [25.8%]; *p* < 0.001) and good neurological outcome at discharge (17 [60.7%], 56 [38.9%], and 3 [2.3%]; *p* < 0.001) between rCAST severity groups. The AUC for the rCAST score in predicting poor neurological outcomes at discharge was 0.859 (95% CI, 0.81–0.90) overall. The AUC was 0.877 (95% CI, 0.82–0.92) in TTM patients and 0.787 (95% CI, 0.69–0.86) in non-TTM patients.

In the univariate logistic regression analysis, age, duration of resuscitation, PLR, rCAST severity, and TTM were associated with a good neurological outcome at discharge (Table 2). Multivariate analysis revealed that age and duration of resuscitation had significantly lower odds for good neurological outcomes at hospital discharge. The PLR (adjusted OR, 6.74; 95% CI, 2.96–15.36) and lower severity of rCAST (high: reference; moderate: adjusted OR, 13.73; 95% CI, 2.99–63.08; low: adjusted OR, 13.86; 95% CI, 3.82–50.22) were associated with higher odds for good neurological outcomes at discharge. TTM remained an important prognostic factor for good neurological outcomes at hospital discharge, even after controlling for other factors (adjusted OR, 3.51; 95% CI, 1.51–8.15).

**Figure 3:**
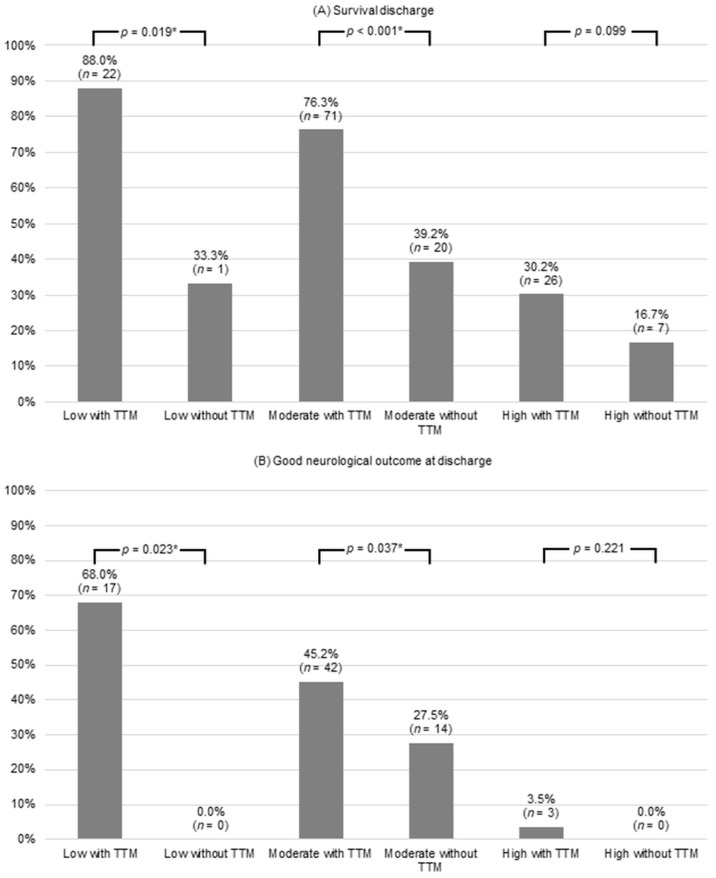
Survival and good neurological outcome at discharge for TTM based on the severity of rCAST. * indicates *p* < 0.05. Abbreviations: TTM, targeted temperature management; rCAST, revised post-cardiac arrest syndrome for therapeutic hypothermia.

As the rCAST severity increased, the discrepancy in the percentage of patients with survival discharge decreased between the TTM and the non-TTM groups (88.0% vs. 33.3%, *p* = 0.019; 76.3% vs. 39.2%, *p* < 0.001; and 30.2% vs. 16.7%, *p* = 0.099, respectively) (Figure 3). The difference in good neurological outcomes at discharge between the groups decreased with increasing rCAST severity (68.0% vs. 0.0%, *p* = 0.023; 45.2% vs. 27.5%, *p* = 0.037; and 3.5% vs. 0.0%, *p* = 0.221, respectively). After adjustment with IPTW for patients in the TTM and non-TTM groups in the moderate severity rCAST group, the baseline and clinical characteristics were well balanced between the groups (Supplementary Table S4). The logistic regression analysis of unadjusted crude and IPTW propensity data in the moderate severity group of rCAST demonstrated that TTM was significantly associated with good neurological outcomes at discharge (Table 3). TTM consistently showed an association with good neurological outcomes at discharge in both the unadjusted crude dataset (OR, 2.18; 95% CI, 1.04–4.55; *p* = 0.039) and the IPTW propensity dataset (OR, 2.31; 95% CI, 1.09–4.91; *p* = 0.029).

## 4. Discussion

We compared the impact of TTM on good neurological outcomes in comatose OHCA survivors stratified by the rCAST score and found that TTM was significantly associated with a good neurologic outcome in the low- and moderate-severity groups.

Lowering body temperatures has a positive impact by decreasing the occurrence of seizures [14,15], reducing cerebral edema [16], alleviating intracranial pressure [17], and minimizing metabolic demand during periods of compromised blood flow [18,19]. However, these benefits may have limited value in patients with low-grade ischemic injury or those who have experienced very severe ischemic injury during cardiac arrest. Several studies have shown that the severity of post-cardiac arrest syndrome (PCAS) may influence the neuroprotective benefit of TTM [5,19,20]. In a study evaluating the differential effects of TTM at 34 °C versus 35–36 °C in PCAS patients, stratified by the presence of hypoxic encephalopathy on early brain imaging. Among patients without hypoxic encephalopathy, those treated with TTM at 34 °C had significantly higher rates of good neurological outcomes compared to those treated at 35–36 °C (68.9% vs. 36.1%, *p* = 0.003). In this subgroup, TTM at 34 °C was associated with better outcomes, with an OR 6.80 (95% CI, 1.19–38.96; *p* = 0.031) [20]. Similarly, a large cohort study of 1319 comatose post-cardiac arrest patients used the Pittsburgh Cardiac Arrest Category (PCAC) to stratify initial illness severity among patients without severe cerebral edema or highly malignant electroencephalogram patterns. The study found that TTM at 33 °C was associated with improved survival and neurological outcomes in patients with the most severe illness, while patients with mild-to-moderate severity had better outcomes with TTM at 36 °C. Notably, those with severe cerebral edema or highly malignant electroencephalogram patterns had poor outcomes, with mortality rates of 98.4% and 96.3%, respectively, regardless of the TTM strategy employed [19]. Our results showed that TTM was associated with good neurological outcomes at discharge in the low- and moderate-severity groups by rCAST classification (68.0% vs. 0.0%, *p* = 0.023; 45.2% vs. 27.5%, *p* = 0.037). However, there was no association between TTM and neurological outcomes in the severe severity group (3.5% vs. 0.0%; *p* = 0.221). While our findings align with previous evidence suggesting a severity-dependent benefit of TTM, there are key differences. Prior studies assessed severity using tools such as early brain imaging for hypoxic encephalopathy, electroencephalogram, or the PCAC score, whereas we used the rCAST score. More importantly, previous studies compared different target temperature strategies among patients who all received TTM. In contrast, our study evaluated outcomes between TTM and non-TTM groups within each severity category, offering a complementary perspective on when TTM would be applied rather than how it is delivered.

The initial development of the post-cardiac arrest syndrome for therapeutic hypothermia score (CAST) aimed to predict the 30-day neurological outcome of patients with PCAS at the ED before the initiation of TTM [21,22]. The uniqueness of the score lies in its specific focus on patients with PCAS undergoing TTM and the fact that its calculation is performed at the ED before the initiation of TTM. However, due to the difficulty in obtaining some of the variables used in the calculation of CAST before initiating TTM and the complexity of the scoring formula, rCAST was developed and validated as a more practical risk classification tool for clinical use [10,23,24,25,26]. Recently, the rCAST score was validated in a US cohort, and it was found that it maintained excellent discrimination for poor neurological outcomes and mortality, regardless of whether patients received TTM treatment [26]. The predictive performance of the rCAST score was better than that of PCAC and similar to previous validation studies, showing a high AUC for predicting outcomes. In a study investigating the relationship between target temperature and neurological outcomes based on the severity of PCAS patients who underwent TTM, it was observed that maintaining a temperature of 33–34 °C in the rCAST moderate severity group resulted in higher rates of good neurological outcomes compared to temperature of 35–36 °C [27]. While the potential utility of the rCAST score in categorizing patients into risk groups is recognized in clinical and research settings, additional investigation is required to ascertain whether the rCAST score should be considered in treatment decisions. Our study examined whether TTM was related to neurological outcomes among comatose OHCA survivors based on the severity and found that TTM was associated with favorable neurological outcomes in patients with low and moderate severity by the rCAST classification, while no association was observed in patients with high severity. Particularly in patients with moderate severity, even after adjusting using IPTW, TTM showed an odds ratio of 2.31 (95% CI, 1.09–4.91) for favorable neurological outcomes. This suggests that TTM could be effective in this particular patient population, and this warrants consideration.

One possible explanation for the observed benefit of TTM in the moderate-severity group is that these patients may have experienced an intermediate level of ischemic brain injury severe enough to require neuroprotection but not so extensive as to be irreversible [20]. In such cases, TTM may help mitigate secondary brain injury by reducing cerebral metabolism, limiting reperfusion-related damage, and preventing further neuronal apoptosis. In contrast, patients with mild injury may already have a good prognosis without aggressive intervention, whereas those with very severe injury may not benefit from TTM due to widespread and irreversible brain damage. Additionally, patients in the high-severity group may be more prone to hemodynamic instability resulting from extensive brain injury, which could be further aggravated by lowering the core temperature, potentially compromising clinical outcomes [28,29]. Therefore, patients in the moderate-severity group may represent an optimal target population for TTM, as their neurological outcomes are more likely to be influenced by timely and targeted intervention.

Despite the usefulness of the rCAST score, efforts should continue to improve early risk stratification tools for assessing the severity of ischemic injury more effectively. These tools should be calculated rapidly and easily within a short period after ROSC. Thus, clinicians should consider incorporating other laboratory or imaging features such as gray/white matter ratio (GWR), which can be easily derived from brain computed tomography results and requires only a slight level of proficiency [30]. GWR has a strong correlation with the severity of brain injury [31,32], thereby significantly enhancing its predictive power to identify patients with such severe brain injury whose prognosis remains poor regardless of any ongoing therapies. Additionally, the combination of other variables, including age, medical history, initial neurological examination findings, and additional laboratory data used in previous studies for neurological prognostic evaluation, may provide valuable insights [33,34]. Further research is needed to determine the optimal combination of these variables and validate its effectiveness.

This study has several limitations. First, this study was a retrospective, observational, registry-based cohort study conducted at a single center, and this may limit the general applicability of the findings and introduce potential biases that cannot be measured or controlled for. Although TTM was generally applied according to a standardized institutional protocol, minor deviations in the temperature control device, target temperature, and duration occurred in some cases, which may have influenced the results. However, prior studies comparing different cooling techniques, including intravascular cooling devices, gel-pad systems, and surface cooling, have reported differences in cooling speed and the precision of temperature maintenance but similar survival rates and neurological outcomes [35,36]. Although the guideline recommends maintaining the target temperature for at least 24 h, some patients in our cohort underwent extended TTM durations based on clinical judgment. While prolonged TTM has been explored in a prior study, evidence regarding its benefit beyond 24 h remains inconclusive [37]. As no consensus exists regarding the optimal TTM approach, the influence of such variations on our findings is likely to be limited. Future research should aim to validate the generalizability of our findings through prospective, multicenter settings with standardized TTM protocols to determine whether similar results can be consistently reproduced. Furthermore, although reduced ICU capacity during the COVID-19 pandemic may have contributed to the inapplicability of TTM in some patients, our data demonstrated that TTM application remained consistent during the pandemic period, suggesting that the clinical care patterns for post-cardiac arrest management remained largely stable, and pandemic-related disruptions had minimal impact on the overall study outcomes. Another aspect to consider is that while TTM was administered in cases that met the indications outlined in the current guidelines, it is possible that TTM was not pursued in some cases due to factors like cost or other considerations. Furthermore, this study included OHCA patients from 2018 to 2023, during which time the guidelines were updated [6,12,38,39,40,41]. These updates have the potential to influence physicians’ clinical decision-making and subsequently affect the outcomes of OHCA survivors. However, there were no changes in the indications, target temperature range, or duration of TTM, and other aspects of post-cardiac arrest care remained largely unchanged during this period. Therefore, these updates are unlikely to have had a significant impact on TTM application or study outcomes. Lastly, this registry focused on neurological outcomes at the time of discharge, thus limiting the ability to assess long-term outcomes.

## 5. Conclusions

Considering the need to identify treatment-responsive patients, the classification of the rCAST score provides valuable insights into the potential benefits of TTM in comatose OHCA survivors. It is crucial to continue investigating and refining risk stratification tools, such as the rCAST score, to optimize the selection of patients who are most likely to benefit from TTM.

## Figures and Tables

**Table 1:** Baseline characteristics of the subjects by rCAST severity groups.

Characteristics	Total (*n* = 300)	Low (*n* = 28)	Moderate (*n* = 144)	High (*n* = 128)	*p*-Value
Age, years	65.5 (51.5–74.00)	64.5 (56.0–72.5)	66.0 (52.0–74.0)	65.5 (50.0–75.0)	0.829
Male	209 (69.7)	21 (75.0)	96 (66.7)	92 (71.9)	0.526
Arrest etiology, trauma	26 (8.7)	0 (0.0)	9 (6.3)	17 (13.3)	0.028 *
Witnessed	225 (75.0)	24 (85.7)	117 (81.3)	84 (65.6)	0.005 *
Bystander CPR	209 (69.7)	20 (71.4)	101 (70.1)	88 (68.8)	0.948
Shockable rhythm	100 (33.3)	20 (71.4)	57 (39.6)	23 (18.0)	<0.001 *
Duration of resuscitation, min	26.5 (13.0–39.0)	12.0 (6.5–16.5)	17.5 (10.0–33.0)	35.5 (27.5–45.5)	<0.001 *
Past medical history					
Hypertension	101 (33.7)	8 (28.6)	54 (37.5)	39 (31.0)	0.432
Diabetes mellitus	86 (28.7)	7 (25.0)	41 (28.5)	38 (30.2)	0.853
Heart disease	82 (27.3)	13 (46.4)	43 (29.9)	26 (20.6)	0.015 *
Stroke	17 (5.7)	0 (0.0)	12 (8.3)	5 (4.0)	0.119
Serum pH	7.014 (6.847–7.162)	7.291 (7.211–7.371)	7.109 (7.022–7.216)	6.847 (6.732–6.938)	<0.001 *
Serum lactate	10.2 (7.4–14.1)	6.8 (3.5–9.5)	8.6 (6.6–12.0)	13.3 (10.1–15.0)	<0.001 *
PLR	133 (44.3)	23 (82.1)	85 (59.0)	25 (19.5)	<0.001 *
Motor score of GCS	1 (1–1)	4 (3–4)	1 (1–1)	1	<0.001 *
TTM	204 (68.0)	25 (89.3)	93 (64.6)	86 (67.2)	0.036 *
Outcome					
Survival to discharge	147 (49.0)	23 (82.1)	91 (63.2)	33 (25.8)	<0.001 *
CPC 1, 2 at discharge	76 (25.3)	17 (60.7)	56 (38.9)	3 (2.3)	<0.001 *

All values are presented as median and interquartile range or as numbers (percentages). * indicates *p* < 0.05. Abbreviations: CPR, cardiopulmonary resuscitation; PLR, pupillary light reflex; GCS, Glasgow coma scale; TTM, targeted temperature management; CPC, cerebral performance category.

**Table 2:** Univariate and multivariate analysis for good neurological outcomes.

Characteristics	Univariate			Multivariate		
	OR	95% CI	*p*-Value	aOR	95% CI	*p*-Value
Age, years	0.98	0.97–1.00	0.021 *	0.97	0.95–0.99	0.010 *
Male	1.19	0.67–2.12	0.553			
Arrest etiology, trauma	0.51	0.17–1.53	0.222			
Witnessed	1.86	0.95–3.61	0.066			
Bystander CPR	1.09	0.62–1.93	0.761			
Duration of resuscitation, min	0.92	0.90–0.95	<0.001 *	0.95	0.92–0.98	<0.001 *
Past medical history						
Hypertension	1.02	0.59–1.77	0.946			
Diabetes mellitus	0.64	0.35–1.18	0.148			
Heart disease	1.20	0.68–2.13	0.535			
Stroke	0.89	0.28–2.83	0.848			
PLR	15.44	7.50–31.90	<0.001 *	6.74	2.96–15.36	<0.001 *
rCAST						
Low	64.39	16.31–254.29	<0.001 *	13.86	3.82–50.25	<0.001 *
Moderate	26.52	8.04–87.43	<0.001 *	13.73	2.99–63.08	0.001 *
High	Reference		<0.001 *	Reference		<0.001 *
TTM	2.56	1.35–4.85	0.003 *	3.51	1.51–8.15	0.003 *

* indicates *p* < 0.05. Abbreviations: OR, odds ratio; CI, confidence interval; aOR, adjusted odds ratio; CPR, cardiopulmonary resuscitation; PLR, pupillary light reflex; rCAST, revised post-cardiac arrest syndrome for therapeutic hypothermia; TTM, targeted temperature management.

**Table 3:** Odds ratio for good neurological outcomes at discharge in the moderate severity group of rCAST.

Dataset	Variable	Good Neurological Outcomes at Discharge		
		OR	95% CI	*p*-Value
Crude	Non-TTM	Reference		
	TTM	2.18	1.04–4.55	0.039 *
IPTW	Non-TTM	Reference		
	TTM	2.31	1.09–4.91	0.029 *

* indicates *p* < 0.05. Abbreviations: OR, odds ratio; CI, confidence interval; TTM, targeted temperature management; IPTW, inverse probability of treatment weighting.

## Data Availability

The data used to support the findings of this study are available from the corresponding author upon reasonable request.

## Supplementary Materials

The following supporting information can be downloaded at: https://www.mdpi.com/article/10.3390/jcm14113931/s1, Supplementary Table S1: Calculation of rCAST; Supplementary Table S2: Comparison of the characteristics between the TTM and non-TTM groups; Supplementary Table S3: Baseline characteristics of the subjects in the pre-pandemic and pandemic periods; Supplementary Table S4: Characteristics of the TTM and non-TTM groups in the moderate severity rCAST group after inverse probability of treatment weighting.

## References

[1] Ichim C., Pavel V., Mester P., Schmid S., Todor S.B., Stoia O., Anderco P., Kandulski A., Müller M., Heumann P. (2024). Assessing Key Factors Influencing Successful Resuscitation Outcomes in Out-of-Hospital Cardiac Arrest (OHCA). J. Clin. Med..

[2] Kim S.-M., Kim Y.-J., Kim Y.-J., Kim W.-Y. (2023). Long-term disability level of 1-month survivors after out-of-hospital cardiac arrest: A nationwide cohort study in Korea using data from 2009 to 2018. Signa Vitae.

[3] Bernard S.A., Gray T.W., Buist M.D., Jones B.M., Silvester W., Gutteridge G., Smith K. (2002). Treatment of comatose survivors of out-of-hospital cardiac arrest with induced hypothermia. N. Engl. J. Med..

[4] Hypothermia after Cardiac Arrest Study Group (2002). Mild therapeutic hypothermia to improve the neurologic outcome after cardiac arrest. N. Engl. J. Med..

[5] Callaway C.W. (2023). Targeted temperature management with hypothermia for comatose patients after cardiac arrest. Clin. Exp. Emerg. Med..

[6] Kim Y.-M., Jeung K.W., Kim W.Y., Park Y.S., Oh J.S., You Y.H., Lee D.H., Chae M.K., Jeong Y.J., Kim M.C. (2021). 2020 Korean guidelines for cardiopulmonary resuscitation. Part 5. Post-cardiac arrest care. Clin. Exp. Emerg. Med..

[7] Sanfilippo F., La Via L., Lanzafame B., Dezio V., Busalacchi D., Messina A., Ristagno G., Pelosi P., Astuto M. (2021). Targeted temperature management after cardiac arrest: A systematic review and meta-analysis with trial sequential analysis. J. Clin. Med..

[8] Kikutani K., Nishikimi M., Shimatani T., Kyo M., Ohshimo S., Shime N. (2021). Differential effectiveness of hypothermic targeted temperature management according to the severity of post-cardiac arrest syndrome. J. Clin. Med..

[9] Kim Y.-J., Kim W.Y. (2021). Cautious application of targeted temperature management in a real-world OHCA population after “TTM2 trial”. Signa Vitae.

[10] Nishikimi M., Ogura T., Nishida K., Takahashi K., Nakamura M., Matsui S., Matsuda N., Iwami T. (2019). External validation of a risk classification at the emergency department of post-cardiac arrest syndrome patients undergoing targeted temperature management. Resuscitation.

[11] Kim B., Kwon H., Kim S.-M., Kim J.-S., Ryoo S.M., Kim Y.-J., Kim W.Y. (2022). Ion Shift Index at the Immediate Post-Cardiac Arrest Period as an Early Prognostic Marker in Out-of-Hospital Cardiac Arrest Survivors. J. Clin. Med..

[12] Nolan J.P., Sandroni C., Böttiger B.W., Cariou A., Cronberg T., Friberg H., Genbrugge C., Haywood K., Lilja G., Moulaert V.R. (2021). European resuscitation council and European society of intensive care medicine guidelines 2021: Post-resuscitation care. Resuscitation.

[13] Cheng K., Wu C., Gu S., Lu Y., Wu H., Li C. (2023). WHO declares the end of the COVID-19 global health emergency: Lessons and recommendations from the perspective of ChatGPT/GPT-4. Int. J. Surg..

[14] Legriel S., Lemiale V., Schenck M., Chelly J., Laurent V., Daviaud F., Srairi M., Hamdi A., Geri G., Rossignol T. (2016). Hypothermia for neuroprotection in convulsive status epilepticus. N. Engl. J. Med..

[15] Bennet L., Dean J.M., Wassink G., Gunn A.J. (2007). Differential effects of hypothermia on early and late epileptiform events after severe hypoxia in preterm fetal sheep. J. Neurophysiol..

[16] Guluma K.Z., Oh H., Yu S.-W., Meyer B.C., Rapp K., Lyden P.D. (2008). Effect of endovascular hypothermia on acute ischemic edema: Morphometric analysis of the ICTuS trial. Neurocrit. Care.

[17] Andrews P.J., Sinclair H.L., Rodriguez A., Harris B.A., Battison C.G., Rhodes J.K., Murray G.D. (2015). Hypothermia for intracranial hypertension after traumatic brain injury. N. Engl. J. Med..

[18] Bacher A., Illievich U.M., Fitzgerald R., Ihra G., Spiss C.K. (1997). Changes in oxygenation variables during progressive hypothermia in anesthetized patients. J. Neurosurg. Anesthesiol..

[19] Callaway C.W., Coppler P.J., Faro J., Puyana J.S., Solanki P., Dezfulian C., Doshi A.A., Elmer J., Frisch A., Guyette F.X. (2020). Association of initial illness severity and outcomes after cardiac arrest with targeted temperature management at 36 °C or 33 °C. JAMA Netw. Open.

[20] Nishikimi M., Ogura T., Nishida K., Takahashi K., Fukaya K., Liu K., Nakamura M., Matsui S., Matsuda N. (2018). Differential effect of mild therapeutic hypothermia depending on the findings of hypoxic encephalopathy on early CT images in patients with post-cardiac arrest syndrome. Resuscitation.

[21] Nishikimi M., Matsuda N., Matsui K., Takahashi K., Ejima T., Liu K., Ogura T., Higashi M., Umino H., Makishi G. (2016). CAST: A new score for early prediction of neurological outcomes after cardiac arrest before therapeutic hypothermia with high accuracy. Intensive Care Med..

[22] Nishikimi M., Matsuda N., Matsui K., Takahashi K., Ejima T., Liu K., Ogura T., Higashi M., Umino H., Makishi G. (2017). A novel scoring system for predicting the neurologic prognosis prior to the initiation of induced hypothermia in cases of post-cardiac arrest syndrome: The CAST score. Scand. J. Trauma. Resusc. Emerg. Med..

[23] Yasuda Y., Nishikimi M., Matsui K., Numaguchi A., Nishida K., Emoto R., Matsui S., Matsuda N. (2021). The rCAST score is useful for estimating the neurological prognosis in pediatric patients with post-cardiac arrest syndrome before ICU admission: External validation study using a nationwide prospective registry. Resuscitation.

[24] Chen C.-H., Wang C.-J., Wang I.-T., Yang S.-H., Wang Y.-H., Lin C.-Y. (2022). Does One Size Fit All? External Validation of the rCAST Score to Predict the Hospital Outcomes of Post-Cardiac Arrest Patients Receiving Targeted Temperature Management. J. Clin. Med..

[25] Misumi K., Hagiwara Y., Kimura T., Hifumi T., Inoue A., Sakamoto T., Kuroda Y., Ogura T. (2023). External validation of the CAST and rCAST score in patients with out-of-hospital cardiac arrest who underwent ECPR: A secondary analysis of the SAVE-J II study. J. Am. Heart Assoc..

[26] Kim N., Kitlen E., Garcia G., Khosla A., Miller P.E., Johnson J., Wira C., Greer D.M., Gilmore E.J., Beekman R. (2023). Validation of the rCAST score and comparison to the PCAC and FOUR scores for prognostication after out-of-hospital cardiac arrest. Resuscitation.

[27] Nishikimi M., Ogura T., Nishida K., Hayashida K., Emoto R., Matsui S., Matsuda N., Iwami T. (2021). Outcome related to level of targeted temperature management in postcardiac arrest syndrome of low, moderate, and high severities: A nationwide multicenter prospective registry. Crit. Care Med..

[28] Delhaye C., Mahmoudi M., Waksman R. (2012). Hypothermia therapy: Neurological and cardiac benefits. J. Am. Coll. Cardiol..

[29] Thomsen J.H., Kjaergaard J., Graff C., Pehrson S., Erlinge D., Wanscher M., Køber L., Bro-Jeppesen J., Søholm H., Winther-Jensen M. (2016). Ventricular ectopic burden in comatose survivors of out-of-hospital cardiac arrest treated with targeted temperature management at 33 °C and 36 °C. Resuscitation.

[30] Kim M.-J., Kim Y.-J., Yum M.-S., Kim W.Y. (2022). Alpha-power in electroencephalography as good outcome predictor for out-of-hospital cardiac arrest survivors. Sci. Rep..

[31] Metter R.B., Rittenberger J.C., Guyette F.X., Callaway C.W. (2011). Association between a quantitative CT scan measure of brain edema and outcome after cardiac arrest. Resuscitation.

[32] Kim S.H., Choi S.P., Park K.N., Youn C.S., Oh S.H., Choi S.M. (2013). Early brain computed tomography findings are associated with outcome in patients treated with therapeutic hypothermia after out-of-hospital cardiac arrest. Scand. J. Trauma. Resusc. Emerg. Med..

[33] Seo D.-W., Yi H., Bae H.-J., Kim Y.-J., Sohn C.-H., Ahn S., Lim K.-S., Kim N., Kim W.-Y. (2021). Prediction of Neurologically Intact Survival in Cardiac Arrest Patients without Pre-Hospital Return of Spontaneous Circulation: Machine Learning Approach. J. Clin. Med..

[34] Kim Y.-J., Kim M.-J., Kim Y.H., Youn C.S., Cho I.S., Kim S.J., Wee J.H., Park Y.S., Oh J.S., Lee D.H. (2021). Background frequency can enhance the prognostication power of EEG patterns categories in comatose cardiac arrest survivors: A prospective, multicenter, observational cohort study. Crit. Care.

[35] Deye N., Cariou A., Girardie P., Pichon N., Megarbane B., Midez P., Tonnelier J.-M., Boulain T., Outin H., Delahaye A. (2015). Endovascular versus external targeted temperature management for patients with out-of-hospital cardiac arrest: A randomized, controlled study. Circulation.

[36] Look X., Li H., Ng M., Lim E.T.S., Pothiawala S., Tan K.B.K., Sewa D.W., Shahidah N., Pek P.P., Ong M.E.H. (2018). Randomized controlled trial of internal and external targeted temperature management methods in post-cardiac arrest patients. Am. J. Emerg. Med..

[37] Kirkegaard H., Søreide E., De Haas I., Pettilä V., Taccone F.S., Arus U., Storm C., Hassager C., Nielsen J.F., Sørensen C.A. (2017). Targeted temperature management for 48 vs 24 hours and neurologic outcome after out-of-hospital cardiac arrest: A randomized clinical trial. JAMA.

[38] Callaway C.W., Donnino M.W., Fink E.L., Geocadin R.G., Golan E., Kern K.B., Leary M., Meurer W.J., Peberdy M.A., Thompson T.M. (2015). Part 8: Post–cardiac arrest care: 2015 American Heart Association guidelines update for cardiopulmonary resuscitation and emergency cardiovascular care. Circulation.

[39] Nolan J.P., Soar J., Cariou A., Cronberg T., Moulaert V.R., Deakin C.D., Bottiger B.W., Friberg H., Sunde K., Sandroni C. (2015). European resuscitation council and European society of intensive care medicine 2015 guidelines for post-resuscitation care. Intensive Care Med..

[40] Kim Y.-M., Park K.N., Choi S.P., Lee B.K., Park K., Kim J., Kim J.H., Chung S.P., Hwang S.O. (2016). Part 4. Post-cardiac arrest care: 2015 Korean guidelines for cardiopulmonary resuscitation. Clin. Exp. Emerg. Med..

[41] Merchant R.M., Topjian A.A., Panchal A.R., Cheng A., Aziz K., Berg K.M., Lavonas E.J., Magid, D.J. on behalf of Adult Basic and Advanced Life Support, Pediatric Basic and Advanced Life Support, Neonatal Life Support, Resuscitation Education Science, and Systems of Care Writing (2020). Part 1: Executive summary: 2020 American Heart Association guidelines for cardiopulmonary resuscitation and emergency cardiovascular care. Circulation.

