# Supplementary material for: Tailoring Targeted Temperature Management in Comatose Out-of-Hospital Cardiac Arrest Survivors: A Retrospective Analysis Based on the rCAST Score Classification
Source: J Clin Med. 2025 Jun 3;14(11):3931. doi: 10.3390/jcm14113931 (PMC12156200; doi:10.3390/jcm14113931)
Supplement: Supplementary file 1 [file jcm-14-03931-s001.zip › jcm-3646852-supplementary.pdf]

Supplementary Table S1. Calculation of rCAST.

Variables	Score			
	0	1	2	3
Initial rhythm	Shockable	Non shockable		
Witness/until ROSC time	<20 min	≤20 min	Unwitnessed	
pH	≥7.31	7.16–7.30	7.01–7.15	≤7.00
Lactate	≤5.0	5.0–10.0	10.1–14.0	≥14.1
Motor score of GCS	≥2	1		
Formula=1.0×(Initial rhythm) + 2.0×(Witness/until ROSC time) + 2.5×(pH) + 0.5×(Lactate) + 4.5×(Motor score of GCS)				

rCAST, revised post-cardiac arrest syndrome for therapeutic hypothermia; ROSC, return of spontaneous circulation; GCS glasgow coma scale.

Supplementary Table S2. Comparison of the characteristics between the TTM and non-TTM groups.

**Characteristics**	**Total** (n = 300)	**TTM** (n = 204)	**Non-TTM** (n = 96)	***p*-value**
**Age, year**	65.5 (51.3–74.0)	66.0 (51.0–74.8)	65.0 (53.0–73.8)	0.94
**Male**	209 (69.7)	146 (71.6)	63 (65.6)	0.30
**Witnessed**	225 (75.0)	158 (77.5)	67 (69.8)	0.15
**Bystander CPR**	209 (69.7)	150 (73.5)	59 (61.5)	0.03*
**Shockable rhythm**	100 (33.3)	73 (35.8)	27 (28.1)	0.19
**Duration of resuscitation, min**	26.5 (13.0–39.0)	26.0 (14.0–38.0)	28.5 (11.0–46.0)	0.30
**Past medical history**				
Hypertension	101 (33.7)	72 (35.3)	29 (30.2)	0.45
Diabetes mellitus	86 (28.7)	60 (29.4)	26 (27.1)	0.76
Heart disease	82 (27.3)	59 (28.9)	23 (24.0)	0.42
Stroke	17 (5.7)	13 (6.4)	4 (4.2)	0.46

**Serum pH**	7.014 (6.847–7.162)	7.007 (6.848–7.168)	7.025 (6.844–7.145)	0.54
**Serum lactate**	10.2 (7.4–14.1)	10.0 (8.1–15.5)	11.4 (8.2–14.9)	0.04*
**PLR**	133 (44.3)	103 (50.5)	30 (31.3)	0.002*
**Motor score of GCS**	1 (1–1)	1 (1–1)	1 (1–1)	0.17
**rCAST score**	13.5 (9.0–16.0)	13.5 (8.1–15.5)	13.5 (10.5–16.0)	0.33
**Outcome**				
Survival to discharge	147 (49.0)	120 (58.8)	27 (28.1)	<0.001*
CPC 1, 2 at discharge	76 (25.3)	62 (30.4)	14 (14.6)	0.003*
**SOFA score at ICU admission**	11 (9–13)	11 (9–12)	11 (9–13)	0.07

All values are presented as median and interquartile range or as numbers (percentages). * indicates $p < 0.05$. Abbreviations: TTM, targeted temperature management; CPR, cardiopulmonary resuscitation; PLR, pupillary light reflex; GCS, glasgow coma scale; rCAST, revised post-cardiac arrest syndrome for therapeutic hypothermia; CPC, cerebral performance category; SOFA, sequential organ failure assessment; ICU, intensive care unit.

Supplementary Table S3. Baseline characteristics of the subjects in the pre-pandemic and pandemic periods.

**Characteristics**	**Pre-pandemic** (*n* = 91)	**Pandemic** (*n* = 209)	***p*-value**
**Age, years**	65.0 (48.0–76.0)	66.0 (53.0–74.0)	0.578
**Male**	58 (63.7)	151 (72.2)	0.140
**Arrest etiology, trauma**	12 (13.2)	14 (6.7)	0.066
**Witnessed**	65 (71.4)	160 (76.6)	0.346
**Bystander CPR**	54 (59.3)	155 (74.2)	0.010*
**Shockable rhythm**	29 (31.9)	71 (34.0)	0.722
**Duration of resuscitation, min**	25.0 (10.0–38.5)	27.0 (14.0–40.0)	0.260
**Past medical history**			

Hypertension	30 (33.3)	71 (34.1)	0.893
Diabetes mellitus	24 (26.7)	62 (29.8)	0.583
Heart disease	31 (34.4)	51 (24.5)	0.078
Stroke	1 (1.1)	16 (7.7)	0.025*
**Serum pH**	7.038 (6.904–7.201)	6.998 (6.838–7.134)	0.195
**Serum lactate**	9.9 (7.5–14.0)	10.8 (7.4–14.0)	0.847
**PLR**	41 (45.1)	92 (44.0)	0.868
**Motor score of GCS**	1 (1–1)	1 (1–1)	0.543
**TTM**	63 (69.2)	141 (67.5)	0.763
**Outcome**			
Survival to discharge	39 (42.9)	108 (51.7)	0.160
CPC 1, 2 at discharge	23 (25.3)	53 (25.4)	0.988
**SOFA score at ICU admission**	11 (9–12)	11 (9–13)	0.600

All values are presented as median and interquartile range or as numbers (percentages). * indicates $p < 0.05$. Abbreviations: CPR, cardiopulmonary resuscitation; PLR, pupillary light reflex; GCS, glasgow coma scale; TTM, targeted temperature management; CPC, cerebral performance category; SOFA, sequential organ failure assessment; ICU, intensive care unit.

Supplementary Table S4. Characteristics of the TTM and non-TTM groups in the moderate severity rCAST group after inverse probability of treatment weighting.

**Characteristics**	**TTM** (n = 93)	**Non-TTM** (n = 51)	**Standard mean difference**
**Age, year**	67 (51–75)	66 (59–71)	-0.0264
**Male**	61 (65.3)	33 (65.5)	-0.0052
**Arrest etiology, trauma**	5 (5.9)	3 (5.9)	-0.0002
**Witnessed**	75 (80.3)	41 (80.8)	-0.012
**Bystander CPR**	65 (69.6)	35 (69.3)	0.0053
**Duration of resuscitation, min**	19 (12–33)	18 (11–36)	-0.0172

**Past medical history**			
Hypertension	35 (37.3)	20 (38.8)	-0.0311
Diabetes mellitus	27 (28.8)	16 (32.2)	-0.0738
Heart disease	29 (31.0)	17 (32.9)	-0.0416
Stroke	7 (8.0)	3 (6.3)	0.0627
**PLR**	55 (58.9)	31 (61.5)	-0.0541

All values are presented as median and interquartile range or as numbers (percentages). Abbreviations: TTM, targeted temperature management; rCAST, revised post-cardiac arrest syndrome for therapeutic hypothermia; CPR, cardiopulmonary resuscitation; PLR, pupillary light reflex.
